# A comparison of the satiating properties of medium-chain triglycerides and conjugated linoleic acid in participants with healthy weight and overweight or obesity

**DOI:** 10.1007/s00394-020-02235-y

**Published:** 2020-04-04

**Authors:** Tyler Maher, Martina Deleuse, Sangeetha Thondre, Amir Shafat, Miriam E. Clegg

**Affiliations:** 1 Diet and Cardiometabolic Health Research Group, Department of Nutritional Sciences, Faculty of Life Sciences and Medicine, King’s College London, London, SE1 9NH UK; 2 Faculty of Health and Life Sciences, Oxford Brookes Centre for Nutrition and Health, Oxford Brookes University, Gipsy Lane, Oxford, OX3 0BP UK; 3 Physiology, School of Medicine, National University of Ireland, Galway, H91 W5P7 Ireland; 4 Department of Food and Nutritional Sciences, University of Reading, Harry Nursten Building, Whiteknights, Reading, RG6 6AP UK

**Keywords:** Appetite, Energy intake, Food intake, Gut-peptide hormones, Ketones, Lipids

## Abstract

**Purpose:**

Inconsistent evidence exists for greater satiety after medium-chain triglycerides (MCT) or conjugated linoleic acid (CLA) compared to long-chain triglycerides (LCT). Furthermore, the mechanisms are poorly understood and effects in people with a healthy weight and those with overweight/obesity have not been compared. This study aimed to compare appetite responses in these groups and examine the mechanisms behind any differences.

**Methods:**

Fifteen participants with healthy weight (BMI: 22.7 ± 1.9 kg·m^−2^) and fourteen participants with overweight/obesity (BMI: 30.9 ± 3.9 kg·m^−2^) consumed a breakfast containing either 23.06 g vegetable oil (CON), 25.00 g MCT oil (MCT), or 6.25 g CLA and 16.80 g vegetable oil (CLA). Appetite, peptide YY (PYY), total ghrelin (TG), *β*-hydroxybutyrate, and gastric emptying (GE) were measured throughout. Energy intake was assessed at an ad libitum lunch and throughout the following ~ 36 h.

**Results:**

Neither MCT nor CLA decreased ad libitum intake; however MCT decreased day 1 energy intake (*P* = 0.031) and the 48-h period (*P* = 0.005) compared to CON. MCT delayed GE (*P* ≤ 0.01) compared to CON, whereas CLA did not. PYY and TG concentrations were not different (*P* = 0.743 and *P* = 0.188, respectively), but MCT increased *β*-hydroxybutyrate concentrations compared to CON (*P* = 0.005) and CLA (*P* < 0.001). *β*-hydroxybutyrate concentrations were higher in participants with overweight/obesity (*P* = 0.009).

**Conclusion:**

Consumption of MCT reduces energy intake in the subsequent 48 h, whereas CLA does not. Delayed gastric emptying or increased *β*-hydroxybutyrate concentrations may mediate this.

## Introduction

Satiety is the process that inhibits further eating after the cessation of an eating episode and is characterised by declined hunger and increased fullness [1]. Satiety enhancement is desirable to consumers as it strengthens internal cues of satiety and can result in calorie reduction without associated feelings of deprivation and hence provide increased dietary compliance [2]. Because dietary fats are the most energy dense macronutrient, fats with functional properties such as enhanced satiety have gained popularity in recent years in response to the continuing prevalence of overweight and obesity [2–5]. ‘Functional fats’ are fats which replace other fats with deleterious effects, or which promote beneficial effects to health [6]. Medium-chain triglycerides (MCT) are the most well-researched and currently popular ‘functional fat’ in terms of satiety, but there are various other fats which also have the potential to beneficially affect weight status and health [7].

MCT have been reported to increase satiety [8–11] and increase energy expenditure [12, 13] compared to more commonly consumed long-chain triglycerides (LCT). This is thought to be achieved through rapid absorption due to the smaller molecular weight of MCT [14], which not only leads to the entirety of the MCT bolus to be absorbed at the point of ingestion (unlike LCT where some remains in the intestine until further consumption [15], but also the production of ketone bodies such as *β*-hydroxybutyrate (*β*-HB) [16], which is thought to be anorexigenic [17]. Other than cholecystokinin (CCK), which has been extensively studied in response to MCT [18–23], the hormonal response to MCT is not well understood. In one study, a MCT preload led to increased peptide YY (PYY) and leptin levels and decreased active ghrelin compared to LCT, but not GLP-1 or total ghrelin (TG) [24]. In another study, although both MCT and LCT increased PYY concentrations, the increase was greater after LCT [25]. Our understanding of the hormonal response to MCT is, therefore, limited.

Conjugated linoleic acid (CLA) is another fat with potential to increase satiety, although evidence for this is limited. The long-term effect of CLA on reducing body fat is widely reported [26–29], and various animal studies have reported decreased intake after CLA supplementation [30–34]. To the author’s knowledge, there is only one study to date that has specifically examined the effect of CLA on satiety in humans [11]. In that study consumption of CLA led to increased satiety compared to LCT, but less than MCT [11]. To date, there is no mechanistic data to support CLA-mediated satiety.

One potential limitation to the literature examining lipids and satiety to date is the lack of participants with overweight and obesity included in studies. It is well known that appetite control is altered with increased adiposity, with attenuated postprandial PYY and ghrelin responses [35] amongst other alterations in appetite hormones. Gastric emptying (GE) has also been shown to be faster in overweight and obesity [36, 37], which may be related to reduced concentrations of pancreatic polypeptide [38]. Therefore, studies examining lipids and satiety should aim to examine the effects in participants with overweight and obesity in order to confirm the findings observed in lean and healthy weight persons are corroborated in this population arguably the ‘target group’ of research aiming to elucidate methods of increasing satiety.

A more complete understanding of the mechanisms behind MCT related satiety, as well as further study into CLA related satiety is warranted. This study was designed to compare the appetite and hormonal responses to MCT and CLA and the effects on subsequent intake, as well as compare the effects in participants of healthy weight to participants with overweight and obesity, which has not previously been investigated. This study also aimed to quantify the GE response to MCT and CLA in comparison to a control lipid in order to evaluate any potential satiety effect caused by differences in GE rates. Therefore, the aim of this study was to compare the satiety response to MCT and CLA in participants with a healthy weight to those with overweight/obesity.

## Methods

### Trial registration

The current study is registered on the ISRCTN clinical trial registry (ISRCTN23021181).

### Design

All participants completed three trials in a random order. They consumed a breakfast smoothie on three non-consecutive days, each of which contained one of the three test oils. ‘Trials’ consist of the standardisation period, day of the laboratory visit and the following day, and these trial periods were separated by a minimum of 48 h and a maximum of 10 days for male participants. For female participants, all trials were conducted during the first 10 days of the menstrual cycle, again with a minimum of 48 h between trials. This was achieved by asking the participant how many days it had been since the start of their menstrual cycle, as indicated by the onset of menstruation, to ensure they were in the follicular phase of their cycle. Female participants were studied across no more than two consecutive menstrual cycles. Participants who were taking progestogen only contraceptive pills were asked to continue taking these throughout the course of the study and were not restricted on dates for testing. After breakfast, data were collected for 3 h, after which participants consumed an ad libitum buffet lunch, and completed weighed food records for the remainder of the day and the following 24 h.

### Participants

Inclusion criteria were as follows: 18–65 years of age, weight stable for the 3 months leading up to the commencement of the study, be taking no medication which could affect appetite, unrestrained eaters and with a BMI of 18.5–24.9 kg·m^−2^ (healthy weight) or a BMI of 25–40 kg·m^−2^ (overweight/obese). Participants with a BMI of 25–30 kg·m^−2^ were also required to have minimum body fat percentages of 25% and 32% (for males and females, respectively) in order to ensure that greater weight was not due to greater muscle mass [39]. After institutional ethical approval (UREC: 171082), participants (healthy weight: M: 10, F: 5, age: 25 ± 5 years, weight: 67.3 ± 9.6 kg, BMI: 22.7 ± 1.9 kg·m^2^, body fat: 18.8 ± 5.5%; overweight/obese: M: 7, F: 7, age: 34 ± 9 years, weight: 91.2 ± 18.2 kg, BMI: 30.9 ± 3.9 kg·m^2^, body fat: 33.1 ± 6.5%) who were recruited through social media, posters and through a research activity mailing list, completed a medical questionnaire and gave their written consent.

### Standardisation

In the 24 h preceding the first trial, participants were required to record all food and drink consumed, along with any physical activity undertaken. This diary was replicated in the 24 h preceding the remaining trials. Strenuous physical activity and alcohol were to be avoided in this 24-h period, and participants were asked to keep caffeine intake at habitual levels.

### Protocol

Participants attended a screening session at the Oxford Brookes Centre for Nutrition and Health, where all experimental trials took place, to determine eligibility. Height was measured (to the nearest 0.1 cm) using a mobile stadiometer (Seca 217, Seca, Birmingham, UK), and body mass and body fat percentage (to the 0.1 kg and 0.1%, respectively) were measured using a body composition monitor (BC-418 MA, Tanita, Amsterdam, the Netherlands). If eligible based on BMI and BF% criteria, participants completed the Three-Factor Eating Questionnaire [40], Dutch Eating Behaviour Questionnaire [41] and Medical History Questionnaire. Participants were provided with commercially available digital weighing scales (Colour Match Digital Scale, Argos, Milton Keynes, UK) and a standardisation booklet, with instructions on how to complete this for the standardisation procedure.

Each trial consisted of one visit to the laboratory, where participants arrived between 7:00 and 9:00 am after a 10–12 h overnight fast. All trials for each participant started at the same time. Participants rested for 10 min in a seated position before a cannula was inserted into an antecubital vein for repeated samples. After baseline samples were taken, participants were provided with the breakfast smoothie which was the vehicle for the experimental oils and were instructed to consume this within 5 min. Immediately upon completion of the breakfast, participants rated the palatability of the drink on visual analogue scales (VAS). Participants then rested for 3 h whilst measures of gastric emptying, subjective sensations of appetite and nausea and blood samples were taken. At 3 h participants consumed an ad libitum buffet lunch until satiation, after which they were free to leave the laboratory. Participants were instructed to complete weighed food diaries for the remainder of the day and for the following 24 h.

### Test breakfast

Participants were provided with 250 mL of a commercially available mango and passion fruit smoothie (Tesco Stores ltd, Cheshunt, UK; 591.9 kJ (139.5 kcal) 0.4 g fat, 32.2 carbohydrate g, 1.5 g protein) to which one of three lipids was added: (1) 23.06 g vegetable oil (Con; rapeseed oil, Tesco Stores Ltd., Cheshunt, UK), (2) 25.00 g MCT oil [MCT; Muscleform, Norfolk, UK, (caproic acid 2%, caprylic acid 50–60%, capric acid 30–45% and lauric acid 3%)] or (3) 6.25 g CLA oil [CLA; USN UK Ltd., Longbridge, UK (5 g of CLA, 50% *c9,t11* isomers and 50% *t10,c12* isomers)] mixed with 16.80 g vegetable oil (rapeseed oil, Tesco Stores Ltd., Cheshunt, UK). MCT are less energy dense than LCT (8.3 kcal/g compared to 9.0 kcal/g) [42] and so the amount of fat added to the smoothie was to achieve an equal energy content across the test meals. The smoothie and test fat were mixed using a food blender for 60 s and were consumed within 5 min. to avoid separation of the fats and were all served at room temperature.

### Gastric emptying

GE was measured by adding 100 mg ^13^C octanoic acid (Eurisotop, France) to the breakfast which resulted in an increase in ^13^CO_2_ in the breath. Octanoic acid is a medium-chain fatty acid which is rapidly oxidised to ^13^CO_2_ after passage through the pyloric sphincter. Breath samples were collected into 12 mL exetainers (Labco, Lampete, UK) in duplicate every 15 min for 3 h. The appearance of ^13^CO_2_ was measured using isotope ratio mass spectrometry (ABCA, Sercon Ltd., Crewe, UK) and results were expressed relative to Vienna Pee Dee Belemnite (V-PBD), an international standard of known ^13^C abundance. Carbon dioxide production rates were estimated to be 5 mmol CO_**2**_·min^−1^·m^−2^ body surface area [43] and body surface area calculated from height and weight according to Haycock et al. [44]*.* The rate of appearance in the breath was used to calculate gastric emptying half time (Thalf) and lag phase (Tlag) according to Ghoos et al. [45] and the latency (Tlat) and ascension (Tasc) according to Schommartz et al. [46]. Thalf is the time required to empty 50% of the ingested meal, Tlat is the initial delay of the cumulative exhalation curve, Tlag is the time between meal ingestion and the start of gastric emptying, and Tasc refers to the high rates of ^13^CO_2_ exhalation between the Tlag and Thalf.

### Subjective sensations

Hunger, fullness, desire to eat (DTE), prospective food consumption (PFC) and nausea were assessed using paper 100-mm visual analogue scales (VAS) where 0 mm indicated ‘not at all’ and 100 mm indicated ‘extremely’. These were recorded at 0 h (baseline), 0.5 h, 1 h, 1.5 h, 2 h, and 3 h. To test the palatability of the breakfasts, participants also rated the appearance, aroma, flavour, pleasantness and texture on 100 mm VAS immediately after the breakfast, with anchors of ‘Do not like at all’ and ‘Like extremely much’ at 0 mm and 100 mm, respectively.

### Blood sampling

Blood was obtained from a superficial antecubital vein via cannulation at 0 h, 0.5 h, 1.0 h, 2.0 h and 3.0 h. Cannulas were kept patent by flushing with 0.9% wt/vol sodium chloride saline (Steripod Normal Saline, Mölnlycke Health Care Ltd, Bedfordshire, UK) immediately after samples. The first 1 mL of each sample was drawn and discarded, after which 6-mL samples were drawn and immediately dispensed into pre-chilled tubes containing K_2_EDTA (Fischer Scientific, Leicester, UK). 1 mL of blood was used for the determination of TG, and the remaining 4 mL was used for the determination of PYY and *β*-HB. The 1 mL aliquot was dispensed into an EDTA tube. All samples were centrifuged for 10 min in a refrigerated (4 °C) centrifuge at 3500*g*. The supernatant of each sample was removed and stored in labelled Eppendorf tubes, and immediately frozen at − 80 °C for later analysis.

Plasma concentrations of *β*-HB (Cayman Chemical, MI, US), PYY (RayBiotech, Cambridge Bioscience, Cambridge, UK) and TG (Merck Millipore, MA, US) were measured by enzyme-linked immunosorbent assay (ELISA). ELISAs were read using a plate reader to quantify absorbance (*β*-HB and PYY: ELx800, BioTek, VT, US; TG: SpectraMax i3x, Molecular Devices, CA, US). Provided quality controls with acceptable high and low ranges ensured precision of analysis. Mean intra-variation of the plates was 5.2% (range: 2.8–10.6%), 7.3% (range: 2.8–13.5%), and 3.9% (range: 1.5–8.8%), for *β*-HB, PYY and TG, respectively.

### Food intake

Three hours after participants consumed the test breakfast, they were presented with an ad libitum pasta lunch made with meat-free Bolognese sauce (Tesco, Cheshunt, UK). In order to ensure each batch of pasta was as closely matched at possible for energy density, pasta was made approximately 1 h prior to serving in identical amounts, in a standardised cooking procedure. Once cooked, 490 g of the Bolognese sauce was mixed thoroughly, before the pasta was distributed into four bowls of approximately 350 g. Immediately before serving, the pasta was reheated for 7 s, cooled for 2 min, then weighed and served. This timing allowed bowls to be replaced when ~ 50–75% of the bowl had been consumed, in order to remove the external cue of an empty bowl terminating the meal. This allowed fresh pasta to be delivered every ~ 180 s, although this was adjusted if the participant was not consuming ~ 50% of the bowl when a fresh bowl was replaced. Participants were explicitly instructed to “eat until you are comfortably full”. Water was consumed ad libitum during the first trial and this amount was repeated in the following trials. Energy intake at the ad libitum meal was calculated from manufacturers packaging and weighed food diaries were analysed using the software package Nutritics Professional (Dublin, Ireland). Participants received training and a detailed instruction booklet on how to complete these food records at the screening session, in order to maximise record accuracy.

### Statistical analysis

Data were analysed using SPSS v.25 software for windows (SPSS inc. Somers, NY, USA). Area under the curve (AUC) values for appetite sensations and for plasma concentrations of hormones vs time curves were calculated using the trapezoidal rule. Plasma hormone concentrations were analysed as raw values and as relative to baseline concentrations (delta). A repeated-measures mixed ANOVA revealed no differences in baseline values for any appetite parameter (all *P* > 0.05), and so a repeated measures mixed ANOVA was run without baseline as a covariate. Repeated measures mixed ANOVAs were used to examine differences between energy intakes between groups at the *ad* libitum lunch, for the rest of the day, the following 24 h and over the 48-h period, as well as to investigate trial order effects. Two-way repeated measures mixed ANOVA were used to examine differences between trials over time for plasma hormone concentrations between groups. Cohen’s *d* was calculated and interpreted according to Cohen [47]. All data were subject to checks for normality using the Shapiro–Wilk test. Where appropriate, post-hoc analyses were conducted using the Bonferroni adjustment. Significance was accepted at the alpha level of *P* < 0.05. Data are expressed as means ± SD unless otherwise stated.

## Results

### Energy intake

There were no differences in food intake at the ad libitum meal, during the rest of the day (from weighed food records), total day 1, day 2 or across the 48-h period between healthy weight and overweight/obese participants (all *P* > 0.05). As such the food intake results are presented for all participants as a whole.

**Fig. 1:**
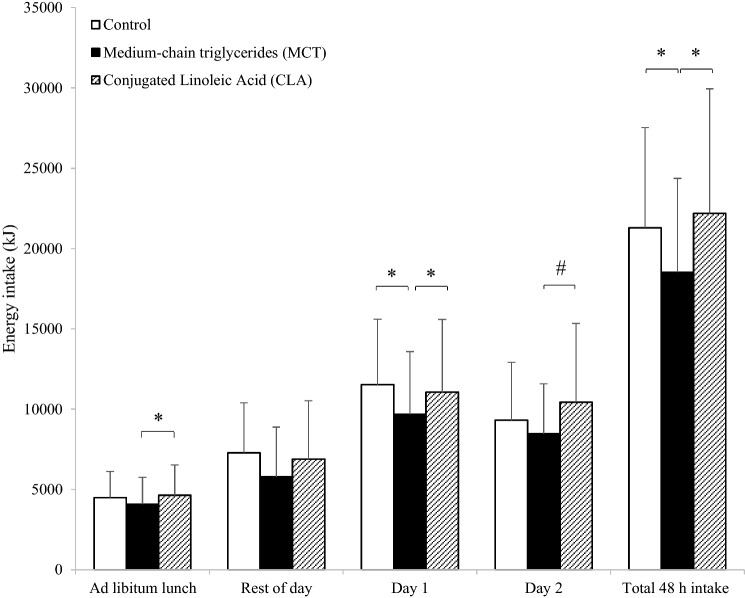
Energy intake (kJ) at the ad libitum lunch, over the course of the trial day (test breakfast, ad libitum lunch and weighed food record), day 2, and over the entire 48-h period. Values are presented as means, with vertical bars representing standard deviation. Asterisk (*) denotes MCT significantly different from both CON and CLA, hash (#) denotes a non-significant trend for lower intake in MCT compared to CLA. Significance is accepted as *P* < 0.05

Energy intake at the ad libitum lunch was significantly different (*F*_(2,54)_ = 3.739, *P* = 0.033, *η*^2^ = 0.122), with decreased intake in MCT (4093 ± 1664 kJ) compared to CLA (4643 ± 1887 kJ, *P* = 0.033, *d* = 0.31). There was no difference between CON (4500 ± 1620 kJ) and MCT (*P* = 0.256) or CLA (*P* = 1.00). There was a trend towards a main effect of trial for the weighed food records, (*F*_(2,50)_ = 3.054, *P* = 0.056, *η*^2^ = 0.109). There was a significant effect of trial on intake over day 1 (*F*_(2,50)_ = 4.855, *P* = 0.012, *η*^2^ = 0.163), as MCT led to lower overall energy intake compared to both CON (9679 ± 3907 kJ vs 11,530 ± 4076 kJ, *P* = 0.031, *d* = 0.46) and CLA (9679 ± 3907 kJ vs 11,057 ± 4540 kJ, *P* = 0.026, *d* = 0.33). There was no difference between CON and CLA (*P* = 1.00). There was a trend for lower energy intake in day 2 (*F*_(2,48)_ = 2.746, *P* = 0.074, *η*^2^ = 0.103), with a tendency for MCT to lead to non-significantly lower intake compared to CLA (8469 ± 3106 kJ vs 10,436 ± 4912 kJ, *P* = 0.072, *d* = 0.49). There was a significant effect of trial on intake over the whole 48-h period (*F*_(2,48)_ = 7.232, *P* = 0.004, *η*^2^ = 0.232), with intakes lower in MCT compared to both CON (18,512 ± 5855 kJ vs 21,307 ± 6222 kJ, *P* = 0.005, *d* = 0.46) and CLA (18,512 ± 5855 kJ vs 22,187 ± 7756 kJ, *P* = 0.005, *d* = 0.54; Fig. 1).

### Appetite sensations

There were no differences between participants with a healthy weight compared to those with overweight/obesity (all *P* > 0.05), so results are presented for the group as a whole. There were no differences between trials for hunger (*F*_(2,54)_ = 0.914, *P* = 0.389, *η*^2^ = 0.033), fullness (*F*_(2,54)_ = 1.793, *P* = 0.176, *η*^2^ = 0.062), DTE (*F*_(2,54)_ = 0.772, *P* = 0.467, *η*^2^ = 0.028), or PFC (*F*_(2,54)_ = 1.060, *P* = 0.342, *η*^2^ = 0.038). There was, however, a significant effect of trial on nausea (*F*_(2, 54)_ = 7.663, *P* = 0.003, *η*^2^ = 0.221), with significant higher AUC values in MCT compared to CON (*P* = 0.007). Analysis of the results indicated this was driven by high nausea ratings for 8 out of 29 participants in MCT.

### Palatability

There was a trend for the MCT breakfast to be more pleasant than CON or CLA, but this was not significant (*F*_(2,56)_ = 2.711, *P* = 0.075, *η*^2^ = 0.088). There were no other differences in any palatability measure (all *P* > 0.192).

### *β-*Hydroxybutyrate

**Fig. 2:**
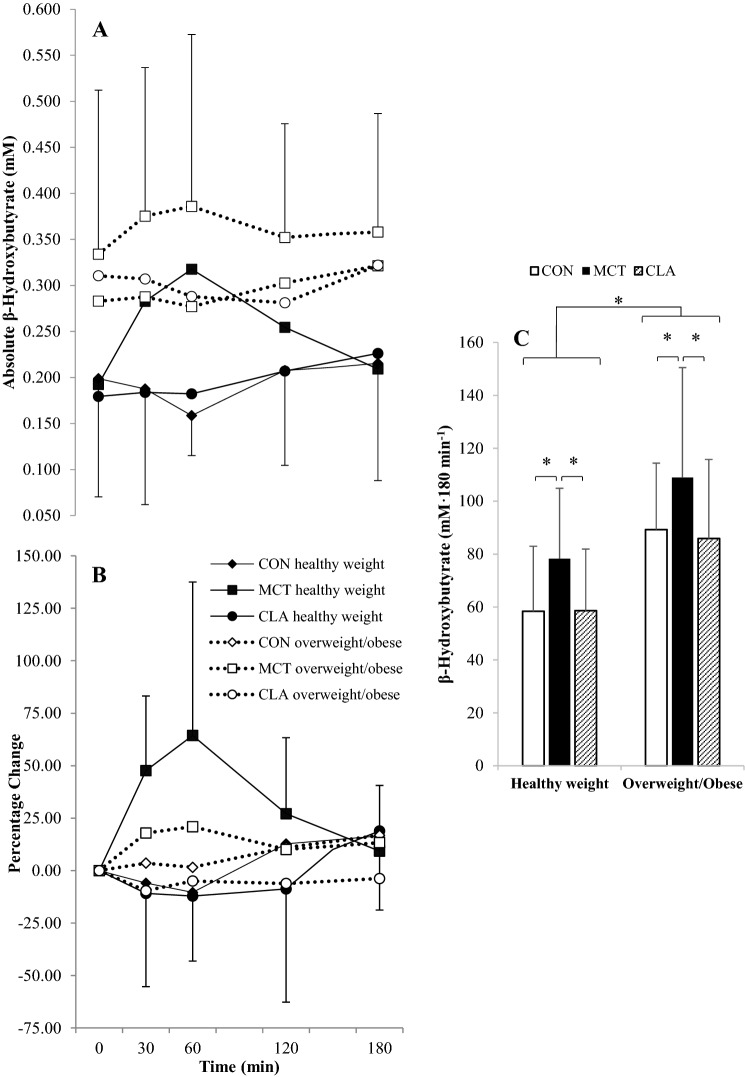
Absolute *β*-HB concentrations (**a**), percentage change from baseline (**b**) and total AUC (**c**) in participants with healthy weight (black symbols; *n* = 15) and overweight/obesity (white symbols; *n* = 14). Data are means with vertical error bars representing SD (some error bars have been removed for clarity). Asterisk (*) denotes significantly different at* P* < 0.05

Baseline concentrations of *β*-HB did not differ between trials (*F*_(2,50)_ = 0.747, *P* = 0.428, *η*^2^ = 0.029). Absolute time-averaged *β*-HB concentrations were significantly higher in the overweight/obese group by 0.110 ± 0.09 mM (*P* = 0.009, *d* = 0.98), but there was no interaction of trial, time and group (*F*_(8,176)_ = 0.649, *P* = 0.586, *η*^2^ = 0.029). There was a significant main effect of trial (*F*_(2,44)_ = 12.810, *P* < 0.001, *η*^2^ = 0.368), and post-hoc tests showed MCT led to greater *β*-HB concentrations compared to both CON (*P* = 0.005, *d* = 0.55) and CLA (*P* < 0.001, *d* = 0.53). There was no main effect of time (*F*_(4,88)_ = 1.216, *P* = 0.311, *η*^2^ = 0.052), but there was a interaction of trial and time (*F*_(8,176)_ = 2.842, *P* = 0.045, *η*^2^ = 0.114), with *β*-HB rising significantly in MCT in the postprandial period compared to CON (Fig. 2a).

There was a significant difference in AUC of *β*-HB concentrations (*F*_(2,50)_ = 17.268, *P* < 0.001, *η*^2^ = 0.409), with significantly higher values in MCT compared to both CON (*P* = 0.001, *d* = 0.60) and CLA (*P* < 0.001, *d* = 0.64). Delta *β*-HB concentrations also followed the same pattern, with significant effects of trial (*F*_(2,48)_ = 8.649, *P* = 0.001, *η*^2^ = 0.265), but not time (*F*_(3,72)_ = 0.292, *P* = 0.831, *η*^2^ = 0.012). Similarly there was a significant interaction of time and trial (*F*_(6,144)_ = 4.084, *P* = 0.012, *η*^2^ = 0.145), as *β*-HB concentrations elevated rapidly to a greater extent in MCT compared to CON or CLA (Fig. 2b).

### PYY

**Fig. 3:**
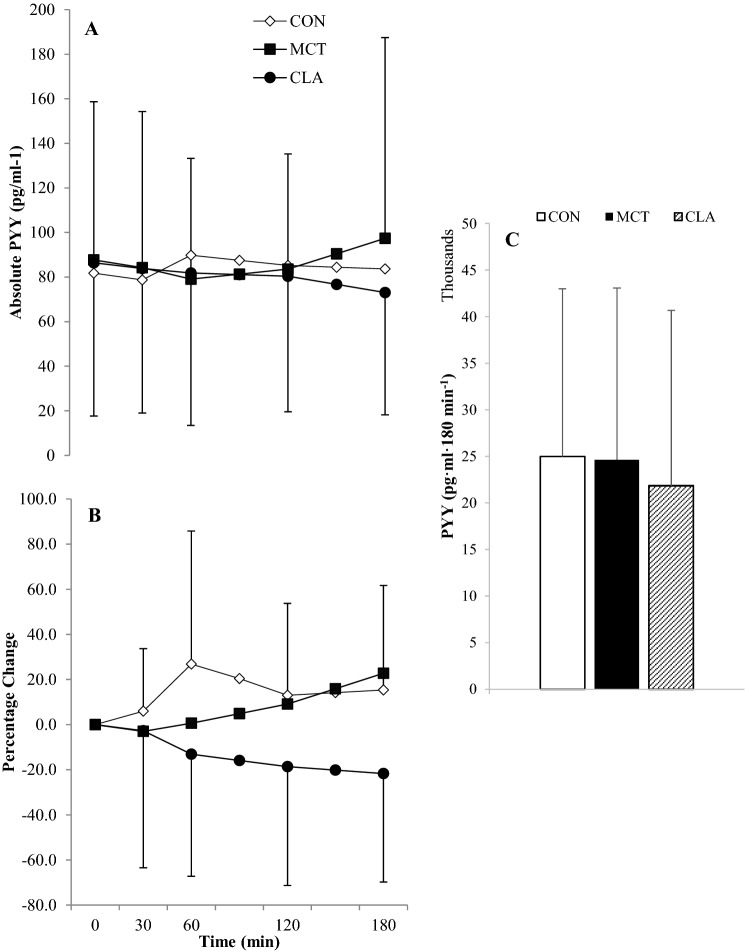
Absolute PYY concentrations (**a**), percentage change from baseline (**b**), and total AUC (**c**) for all participants (*n* = 29). Data are means with vertical error bars representing SD (some error bars have been removed for clarity)

Baseline concentrations of PYY did not differ between trials (*F*_(2,44)_ = 2.150, *P* = 0.145, *η*^2^ = 0.089). PYY was also not affected by group (*P* = 0.170), and thus data are shown for all participants as a whole group. There were no main effects for trial (*F*_(2,44)_ = 0.235, *P* = 0.743, *η*^2^ = 0.011) or time (*F*_(4,88)_ = 0.373, *P* = 0.765, *η*^2^ = 0.017). There was a trend for an interaction between trial and time, as PYY concentrations increased postprandially in CON, peaking at 60 min and decreasing thereafter, whereas PYY levels decreased until 60 min in MCT, then began to rise, and PYY decreased throughout the whole 180 min in CLA. This interaction was non-significant (*F*_(8,176)_ = 2.136, *P* = 0.099, *η*^2^ = 0.088; Fig. 3a). There was no difference between trials for PYY AUC (*P* = 0.647). Delta PYY concentrations showed no effect for trial, despite a trend for suppressed concentrations in CLA compared to both other trials (*F*_(2,46)_ = 2.962, *P* = 0.078, *η*^2^ = 0.114) or time (*F*_(3,69)_ = 0.723, *P* = 0.542, *η*^2^ = 0.030). There was, however, a significant interaction of time and trial (*F*_(6,138)_ = 3.458, *P* = 0.012, *η*^2^ = 0.131), with delta PYY peaking quickest in CON at 60 min, whereas delta PYY in MCT slowly increased after an transient decrease, and CLA continually decreased throughout the whole 180 min (Fig. 3b).

### Total ghrelin

**Fig. 4:**
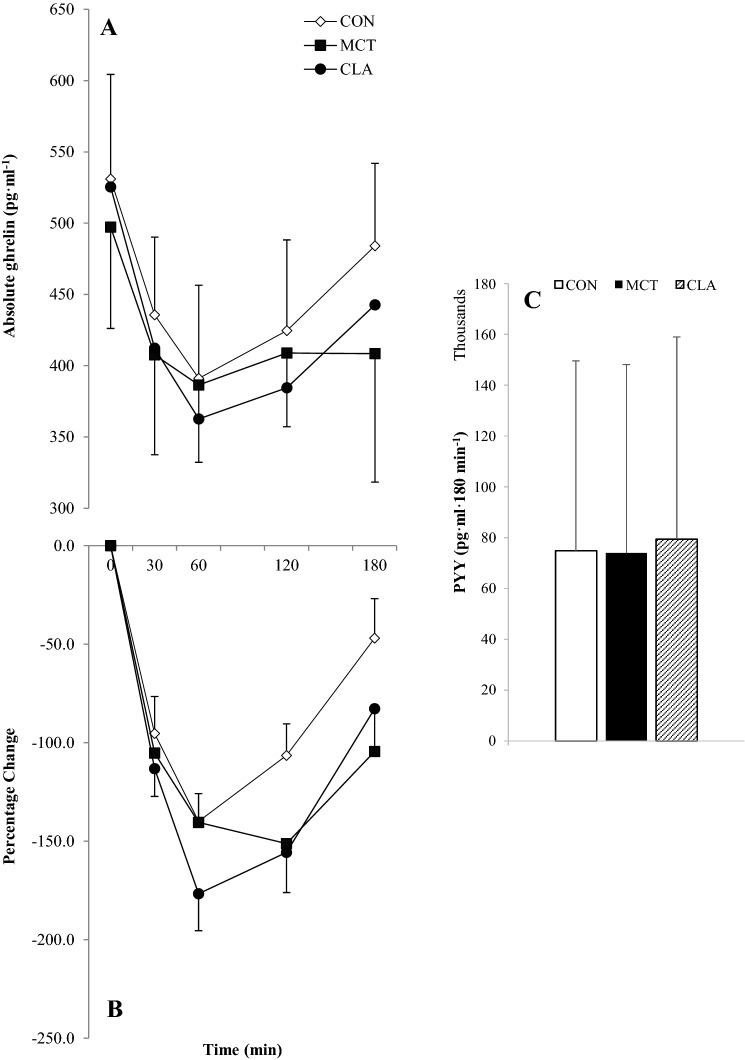
Absolute total ghrelin concentrations (**a**), percentage change from baseline (**b**) and total AUC (**c**) for all participants (*n* = 29). Data are means with vertical error bars representing SD (some error bars have been removed for clarity)

There was no difference between participants with a healthy weight and those with overweight/obese for absolute TG concentrations (*F*_(1,17)_ = 1.112, *P* = 0.306, *η*^2^ = 0.061), and 3-way interaction of trial, time and group (*F*_(8,136)_ = 1.037, *P* = 0.396, *η*^2^ = 0.058), and so results are presented for the data as a whole. There were no main effects for trial (*F*_(2,36)_ = 1.786, *P* = 0.188, *η*^2^ = 0.090) but there was a main effect of time (*F*_(4,72)_ = 19.678, *P* < 0.001, *η*^2^ = 0.522). TG concentrations dropped postprandially, reaching nadir at 90 min. Post-hoc tests revealed all time points were significantly lower than baseline other than 180 min (all *P* < 0.01), where concentrations increased to non-significant levels. There was no interaction of time and trial (*F*_(8,144)_ = 1.827, *P* = 0.131, *η*^2^ = 0.092; Fig. 4a). A repeated measures ANOVA revealed a significant change from baseline TG concentrations (*F*_(2,48)_ = 3.511, *P* = 0.038, *η*^2^ = 0.128). Post-hoc tests showed that values were tended to be lower in MCT compared to CON (*P* = 0.06, *d* = 0.23), but there was no difference between CLA and CON (*P* = 0.575), or MCT and CLA (*P* = 0.496). Delta TG followed a similar pattern as absolute values, as there were no main effects for trial, although this approached significance (*F*_(2,40)_ = 3.119, *P* = 0.055, *η*^2^ = 0.135) as was the same for the interaction of trial and time (*F*_(6,120)_ = 2.107, *P* = 0.087, *η*^2^ = 0.095). There was, however, a significant main effect of time (*F*_(3,60)_ = 16.265, *P* < 0.001, *η*^2^ = 0.449), with delta concentrations decreasing to a similar extent below baseline in all trials (Fig. 4b).

### Gastric emptying

There was no effect of group on Thalf (*P* = 0.939), Tlag (*P* = 0.463), Tlat (*P* = 0.267) or Tasc (*P* = 0.842), and so gastric emptying parameters are presented for all participants as a whole group. There were significant effects of trial on Thalf (*F*_(2,42)_ = 15.906, *P* < 0.001, *η*^2^ = 0.431), Tlag (*F*_(2,42)_ = 29.120, *P* < 0.001, *η*^2^ = 0.581), Tlat (*F*_(2,42)_ = 10.120, *P* < 0.001, *η*^2^ = 0.325) and Tasc (*F*_(2,42)_ = 15.502, *P* < 0.001, *η*^2^ = 0.425). Post-hoc tests showed MCT delayed all GE parameters compared to both CON and CLA (Thalf: *P* = 0.001, *d* = 1.37; *P* = 0.001, *d* = 1.12; Tlag: *P* < 0.001, *d* = 1.68; *P* < 0.001, *d* = 1.49; Tlat: *P* = 0.01, *d* = 0.95; *P* < 0.001, *d* = 1.24; Tasc: *P* = 0.001, *d* = 1.39; *P* = 0.002, *d* = 1.08). There were no differences between CON and CLA (all *P* = 1.00). There was also no interaction of trial and group in Thalf (*F*_(2,42)_ = 0.2001, *P* = 0.164, *η*^2^ = 0.087) or Tasc (*F*_(2,42)_ = 1.757, *P* = 0.196, *η*^2^ = 0.077), Table 1.

**Table 1:** Gastric emptying (GE) half time, lag phase, latency phase and ascension time of all participants (*n* = 29)

GE parameter	CON		MCT		CLA	
	Total		Total		Total	
Half time (min)	121.8 ± 46.5		269.8 ± 168.9*		131.4 ± 78.3	
Lag phase (min)	44.8 ± 16.5		77.7 ± 22.6*		45.3 ± 20.7	
Latency phase (min)	36.8 ± 15.4		51.1 ± 14.9*		34.7 ± 11.5	
Ascension time (min)	165.6 ± 40.2		317.0 ± 177.6*		178.4 ± 79.3	

	Healthy-weight	Overweight/obese	Healthy-weight	Overweight/obese	Healthy-weight	Overweight/obese
Half time (min)	105.7 ± 43.7	136.6 ± 45.4	308.5 ± 204.1	228.0 ± 114.1	114.5 ± 46.0	149.7 ± 101.8
Lag phase (min)	38.5 ± 16.2	50.6 ± 15.1	83.2 ± 25.3	71.7 ± 18.5^#^	41.4 ± 16.2	49.6 ± 24.7
Latency phase (min)	32.1 ± 10.4	41.1 ± 18.2	54.1 ± 18.3	47.9 ± 9.7^#^	32.9 ± 10.7	36.7 ± 12.5
Ascension time (min)	152.3 ± 43.0	177.9 ± 34.3	357.4 ± 211.1	273.5 ± 126.9	161.3 ± 44.4	197.0 ± 104.1

Values are means ± SD

*CON* control trial, *MCT* medium-chain triglyceride trial, *CLA* conjugated linoleic acid trial

*Significantly different from both CON and CLA

^#^Significant interaction of trial and group. Significance accepted at the* P* < 0.05 level

### Trial order effects

There were no trial order effects for energy intake for the group as a whole (*P* > 0.05, *η*^2^ > 0.030) or when accounting for participants with healthy weight or overweight/obesity (*P* > 0.05, *η*^2^ > 0.039). GE followed this pattern, with no evidence of a trial order effect for the group as a whole (*P* > 0.05, *η*^2^ > 0.050) or for when accounting for weight status (*P* > 0.05, *η*^2^ > 0.026).

## Discussion

The primary finding of this study was that MCT did not reduce energy intake at the first meal after their consumption, but reduced energy intake over the following 48 h compared to LCT. This was not affected by weight status, and appears to be mediated by a delaying of GE, an increase in the anorexigenic ketone body *β*-HB, or a combination of the two. It is possible the tendency for decreased energy intake at the ad libitum lunch was caused by *β*-HB which then contributed to the decrease in overall energy intake. Conversely, CLA does not alter energy intake in comparison to a LCT control.

The literature surrounding MCT and satiety is equivocal, with studies reporting decreased energy intake after MCT [8–10, 48] and others reporting no effect [49, 50]. The results of the current study corroborate previous findings from our laboratory [11] which found no difference in energy intake at the first meal post consumption of MCT and LCT, but decreased energy intake in later eating episodes and overall energy intake throughout the course of the day after MCT consumption. However, in our previous study, CLA also reduced energy intake to a similar extent, whereas the results of the current study do not support that finding. To our knowledge, only our previous study has investigated the acute effect of CLA on satiety and energy intake in humans, although CLA has been shown to decrease feed intake in mice [33], which is reportedly due to suppressed expression of the potent orexigenic neurons neuropeptide Y and Agouti-related peptide [34]. In the CLA arm of the current study, participants consumed a mixture of CLA and LCT. This dosage was based on our previous work which used the same dosage in the same vehicle (breakfast smoothie) [11]. Information regarding the composition of the CLA (i.e. which isomers in what ratio) was not available in the previous study, and thus it is possible that the CLA given in that study is different to the one given in the current study. Due to its effect on gene expression relating to the regulation of triglyceride storage, the *t10,c12* isomer is reported to exert the greatest anti-adipogenic effect [51], but whether this also relates to acute satiety is unknown. In the current study, participants ate the ad libitum lunch at a fixed timepoint, 180 min after consumption of the breakfast. In the previous study, participants were to request the lunch, and CLA led to a significantly delayed time-to-meal request than the control [11]. On the basis that the ad libitum lunch was later in the day, this may partly explain why there was reduced intake later in the day compared to the control in that study, whereas we report no difference in the current study. Due to the distinct lack of research examining CLA and satiety, it is difficult to draw clear conclusions, but this study does not support the finding that CLA reduces energy intake.

The mechanisms behind MCT-enhanced satiety are still under debate. Early research has shown that the absorption of MCT is quicker than LCT, bypassing the lymphatic system and traveling directly to the liver via the portal vein, where they undergo *β*-oxidation [52]. However, we show delayed GE after MCT compared to LCT. This is in contrast with previous studies showing that MCT do not stimulate CCK secretion (a potent fat-related inhibitor of GE) [53] or accelerate small-bowel transit time [54]. These findings are also disparate to previous research showing that gastric emptying was accelerated following MCT (compared to carbohydrate which is known to empty faster than LCT) [55]. However, we have now seen delays in gastric emptying compared to LCT across several studies [56]. MCT may delay GE due to a higher osmolarity than LCT, which is well known to be inversely related to GE [57]. GE and hunger are known to be correlated [58], so slower GE may be the primary mechanism by which satiety is induced on the MCT trial. Our results also indicate MCT lead to the production of the ketone body *β*-hydroxybutyrate, which is thought to possess anorexigenic properties via a glucose-sparing mechanism and via central action in the brain [17, 59, 60]. Decreases of blood glucose have been shown to promote hunger in order to promote food intake and prevent hypoglycaemia [61], and thus if *β*-hydroxybutyrate attenuates a decrease in blood glucose, hunger should also be attenuated. *β*-hydroxybutyrate rose significantly in response to MCT ingestion in the current study, yet returned to levels similar to baseline by the end of the data collection period, similarly to previous findings investigating the ketone body [60]. Therefore, suppressed energy intake later in the day is unlikely to be mediated by this mechanism.

St-Onge et al. [24] reported no differences in TG concentrations after meals containing MCT or LCT, but greater suppression of acylated ghrelin in LCT. They also report a greater postprandial rise in PYY after MCT compared to LCT. Our results support these findings, as we report no difference in TG concentrations after breakfasts containing LCT, MCT or CLA. Our findings do not, however, reciprocate those regarding PYY, as we found no difference in absolute PYY concentrations and a significantly greater increase from baseline at 60 min in LCT. However, whereas PYY concentrations peaked at 60 min and started to decrease in LCT, concentrations continued to rise in MCT, and failed to reach a plateau. It is possible that PYY levels may have increased further and remained elevated, as it has been shown previously that PYY can remain elevated for beyond 300 min [62], which may explain decreased intake later in the day and in the following 24 h. As PYY secretion is initiated by the sensing of nutrients in the gastrointestinal lumen [63], this may be linked to the delay in gastric emptying caused by MCT. Further work is needed, utilising a longer sampling period, in order to investigate these speculations. Neary et al. [64] reported that only acylated ghrelin exerts orexigenic effects as only the acylated form can bind to the growth hormone secretagogue receptor type 1a (GHS-R1a) in order to activate the lateral hypothalamus: the orexigenic centre of the brain. More recent evidence has suggested that lipids suppress total and acylated ghrelin to the same extent [65], and thus TG may act as a surrogate marker for acylated ghrelin. Regardless, future work should include acylated ghrelin analysis to confirm this.

This study is, to our knowledge, the first study to compare the effect of MCT and CLA in healthy weight to overweight/obese individuals. Increased body fat has been shown to decrease circulating PYY concentrations as well as attenuated postprandial suppression of ghrelin [66]. Studies examining appetite regulation should take differences in these populations into consideration, as findings in lean or healthy weight individuals may not represent those with increased body mass or adiposity. We report no differences in energy intake between individuals with healthy weight and individuals with overweight or obesity; thus MCT exert same effect in both groups. This is despite our reporting increased *β*-HB and accelerated Tlag and Tlat in overweight/obese participants after MCT ingestion, where these parameters were delayed further in overweight/obese participants in LCT and CLA.

This study has shown novel findings that MCT exerts similar effects in healthy weight and obese individuals, via a MCT-mediated delay in gastric emptying, elevation in *β*-HB concentrations and suppression of ghrelin concentrations to a similar extent as LCT. However, there are several limitations to consider. We found that nausea was significantly higher in the MCT trial compared to both CON and CLA. This confounds our findings, as decreases in energy intake may be mediated by nausea, rather than altered appetite regulation; which would discredit the use of MCT. However, the differences in nausea scores were driven by high nausea in a small number (8/29) of participants. Why MCT causes nausea in some but not others is currently unknown and if MCT are to be developed or promoted as a satiating food product, which must be investigated to ensure their tolerability first. Practically, a product which causes nausea is unlikely to gain popularity or be utilised chronically, which would be the aim if they were designed to be utilised in weight loss strategies. Our results indicate most individuals do not respond negatively to MCT ingestion and that adverse effects are not related to palatability as this tended to be higher for the MCT. We also examined TG and not acylated (active) ghrelin, as discussed above.

In conclusion, we have shown that a single feeding of MCT reduce energy intake over a 48-h period compared to equicaloric LCT, whereas CLA do not. This may be mediated by increased *β*-HB concentrations or via delayed gastric emptying, which in turn may lead to prolonged elevated PYY concentrations; although further work is needed to confirm these hypotheses. We also show that MCT exert a similar effect in participants with healthy weight and overweight or obesity.
